# Characterization of an immortalized human small airway basal stem/progenitor cell line with airway region-specific differentiation capacity

**DOI:** 10.1186/s12931-019-1140-9

**Published:** 2019-08-23

**Authors:** Guoqing Wang, Howard H. Lou, Jacqueline Salit, Philip L. Leopold, Sharon Driscoll, Juergen Schymeinsky, Karsten Quast, Sudha Visvanathan, Jay S. Fine, Matthew J. Thomas, Ronald G. Crystal

**Affiliations:** 1 Department of Genetic Medicine, Weill Cornell Medical College, 1300 York Avenue, Box 164, New York, NY 10065 USA; 2 Boehringer Ingelheim Pharma GmbH & Co. KG, Biberach an der Riss, Germany; 3 Boehringer Ingelheim Pharmaceuticals, Ridgefield, CT USA

**Keywords:** Small airway, Basal cells, Immortalization, Telomerase, Single cell

## Abstract

**Background:**

The pathology of chronic obstructive pulmonary disease (COPD), idiopathic pulmonary fibrosis (IPF) and most lung cancers involves the small airway epithelium (SAE), the single continuous layer of cells lining the airways ≥ 6th generations. The basal cells (BC) are the stem/progenitor cells of the SAE, responsible for the differentiation into intermediate cells and ciliated, club and mucous cells. To facilitate the study of the biology of the human SAE in health and disease, we immortalized and characterized a normal human SAE basal cell line.

**Methods:**

Small airway basal cells were purified from brushed SAE of a healthy nonsmoker donor with a characteristic normal SAE transcriptome. The BC were immortalized by retrovirus-mediated telomerase reverse transcriptase (TERT) transduction and single cell drug selection. The resulting cell line (hSABCi-NS1.1) was characterized by RNAseq, TaqMan PCR, protein immunofluorescence, differentiation capacity on an air-liquid interface (ALI) culture, transepithelial electrical resistance (TEER), airway region-associated features and response to genetic modification with SPDEF.

**Results:**

The hSABCi-NS1.1 single-clone-derived cell line continued to proliferate for > 200 doubling levels and > 70 passages, continuing to maintain basal cell features (TP63^+^, KRT5^+^). When cultured on ALI, hSABCi-NS1.1 cells consistently formed tight junctions and differentiated into ciliated, club (SCGB1A1^+^), mucous (MUC5AC^+^, MUC5B^+^), neuroendocrine (CHGA^+^), ionocyte (FOXI1^+^) and surfactant protein positive cells (SFTPA^+^, SFTPB^+^, SFTPD^+^), observations confirmed by RNAseq and TaqMan PCR. Annotation enrichment analysis showed that “cilium” and “immunity” were enriched in functions of the top-1500 up-regulated genes. RNAseq reads alignment corroborated expression of CD4, CD74 and MHC-II. Compared to the large airway cell line BCi-NS1.1, differentiated of hSABCi-NS1.1 cells on ALI were enriched with small airway epithelial genes, including surfactant protein genes, LTF and small airway development relevant transcription factors NKX2–1, GATA6, SOX9, HOPX, ID2 and ETV5. Lentivirus-mediated expression of SPDEF in hSABCi-NS1.1 cells induced secretory cell metaplasia, accompanied with characteristic COPD-associated SAE secretory cell changes, including up-regulation of MSMB, CEACAM5 and down-regulation of LTF.

**Conclusions:**

The immortalized hSABCi-NS1.1 cell line has diverse differentiation capacities and retains SAE features, which will be useful for understanding the biology of SAE, the pathogenesis of SAE-related diseases, and testing new pharmacologic agents.

**Electronic supplementary material:**

The online version of this article (10.1186/s12931-019-1140-9) contains supplementary material, which is available to authorized users.

## Introduction

The small airway epithelium (SAE), comprised of basal, intermediate, club, mucous and ciliated cells, plays a central role in the pathogenesis of chronic obstructive pulmonary disease (COPD), idiopathic pulmonary fibrosis (IPF), cystic fibrosis (CF) and most lung cancers [1–6]. In humans, the basal cells (BC) function as the SAE stem/progenitor for the intermediate and differentiated club, mucous and ciliated cells [7–12]. Based on methods to sample SAE and to isolate BC from the SAE brushes obtained by fiberoptic bronchoscopy [9, 12–14], we have characterized SAE and SAE BC in normal non-smokers, asymptomatic smokers, and individuals with COPD using mRNA microarrays, RNAseq, single cell transcriptome, methylation, microRNA, metabolome and protein levels, characterizing the BC in health and dysregulation of the BC population associated with smoking and COPD [13–20].

While isolation of human SAE and SAE BC by bronchoscopy and brushing permits assessment of primary BC, the procedure is invasive, time consuming and expensive, and the primary BC can be cultured only for 3 to 4 passages before becoming senescent. In this context, if it is possible to immortalize normal human SAE BC that retain the capacity to differentiate to ciliated, secretory, and other differentiated cell types in vitro on air-liquid interface (ALI) culture, it would be very useful to the investigation of SAE biology in health and disease, and the assessment of pharmacologic agents targeted to modify dysregulated BC biology relevant to the pathogenesis of human lung disease. In the current study, we have generated hSABCi-NS1.1, an immortalized human small airway basal cell line from the brushed epithelium of a healthy non-smoker. hSABCi-NS1.1 cells can be passaged for at least 200 population doublings, have the capacity to differentiate into the major differentiated SAE club, mucous and ciliated cells, as well as rare SAE cell types, including surfactant protein positive cells and novel ionocytes, and have the capacity to recapitulate SAE disease-relevant biology when stressed with relevant signals.

## Methods

### Study population and biologic samples

The selected donor was a 50 yr. old African American male healthy non-smoker recruited under a protocol approved by the Weill Cornell Medical College Institutional Review Board. The subject was characterized as phenotypically normal on the basis of clinical history and physical examination, routine blood screening tests, urinalysis, chest X-ray, ECG and pulmonary function testing. The non-smoking status was confirmed by history, venous carboxyhemoglobin levels and urinalysis for levels of nicotine (< 2 ng/ml) and its derivative cotinine (< 5 ng/ml). Following written informed consent, bronchoscopy was used to collect the small airway epithelium (SAE) as previously described [13, 17, 20]. Part of the brushed SAE was used for genome-wide expression assessment by microarray (Affymetrix Human Genome U133 Plus 2.0 arrays, Santa Clara CA). Basal cells (BC) were purified from the remainder of the brushed small airway epithelium by trypsinization and selective culturing of BC on collagen-coated tissue culture flasks [9, 12]. On a morphologic level, these cells do not have motile cilia or mucous granules. The cells were expanded in vitro for one passage and stored in the liquid nitrogen for further use. A subset of published microarray dataset [20] from matched trachea, large (3rd -4th order bronchi) and small airway epithelium (10th to 12th order bronchi) of 9 healthy nonsmoker subjects was used as reference to confirm the expression pattern of donor SAE from current study. The gene expression pattern was compared by unsupervised-cluster analysis.

### Immortalization

The primary small airway BC were cultured in flasks coated with human type IV collagen (Sigma-Aldrich, St. Louis, MO) and supplied with Small Airway Epithelial Cell Growth Medium (SAGM; Lonza, Basel, Switzerland). Passage 1 small airway BC cells from the selected donor were immortalized using retrovirus-mediated expression of human telomerase reverse transcriptase (hTERT) with a puromycin resistance selection marker, as previously described [21]. The parental immortalized small airway cell line was isolated using puromycin selection for 10 days. The surviving cell line was termed as human small airway basal cell immortalized-nonsmoker 1 (hSABCi-NS1). To isolate a single-cell clone, the parental hSABCi-NS1 cell was serially diluted into 10 cm dishes without collagen coating to facilitate cell clone formation [22]. After seeding, the clones were marked and monitored daily to make sure each cell cluster was single-cell-derived. We observed that different cell clones demonstrated different biological features. After 12 days, relatively large clones were picked using sterile cloning cylinders and expanded in type IV collagen-coated flasks with SAGM. One single-cell-clone, termed hSABCi-NS1.1, was expanded and characterized. The morphology of the basal cells in culture over time was documented by an Olympus IX71 inverted microscope with an Olympus DP73 camera (New Jersey Scientific Inc., Somerset, NJ). The cell numbers of each passage were counted by a Countess automated cell counter (Thermo-Fisher, Waltham, MA). Since passage 5 of the hSABCi-NS1.1 culture, the cells were maintained in T25 flasks with type IV collagen. The cells were switched to PneumaCult Ex Plus medium (Stemcell Technologies, Cambridge, MA) from passage 40 onward. The population doubling level and cell doubling time were calculated according to ATCC Animal Cell Culture Guide (https://www.atcc.org/). Additional quality control was performed on passage 46 hSABCi-NS1.1 cells which had been stored in the liquid nitrogen for 131 days. As a control for comparison, a new vial of passage 2 primary parental cells was thawed and cultured under the same culture conditions (PneumaCult Ex plus medium; Stemcell Technologies).

### Air-liquid Interface culture

The basal cell line was seeded at a density of 1.5 × 10^5^ cells/100 μl/well onto a Transwell insert (0.4 μm size pore; 0.3 cm^2^ well; Corning, Corning, NY) coated with human type IV collagen (Sigma-Aldrich, St. Louis, MO) in PneumaCult Ex Plus medium (Stemcell Technologies). The lower chamber contained 0.5 ml PneumaCult Ex Plus medium. After 1 day, media in both upper and lower chambers were replaced with fresh Ex Plus medium. Two days post seeding, the media in the lower chamber was replaced with PneumaCult-ALI maintenance media (Stemcell Technologies). The media in the upper chamber was removed to expose the apical surface to air and establish the air-liquid interface (ALI; day of medium removal referred to as ALI day 0). The ALI cultures were then grown at 37 °C, 5% CO_2_, with fresh PneumaCult-ALI media changes every 2 to 3 days. The apical surface was washed with 1x PBS once a week to remove accumulated mucus. Transepithelial electrical resistance (TEER) was measured with a Millicell ERS-2 Voltohmmeter (Millipore, Burlington, MA). For each time point, 3 independent wells/group were measured.

### Immunofluorescence staining

Cells were assessed in chamber slides (Thermo-Fisher) or on ALI wells, fixed in 4% paraformaldehyde diluted in PBS from a 16% aqueous stock (Electron Microscopy Science, Hatfield, PA) for 20 min and washed with PBS. Standard immunofluorescence staining methods were used [22, 23]. The primary antibodies were rat anti-SCGB1A1 (MAB4218, R&D, Minneapolis, MN); mouse anti-TP63 (sc-8431, Santa Cruz Biotechnology, Dallas, TX), mouse anti-KRT14 (C8791, Sigma-Aldrich, St. Louis, MO), mouse anti-TJP1 (33-9100, Thermo-Fisher), mouse anti-CHGA (MA5-13096, Thermo-Fisher), mouse anti-MUC5AC (VP-M657, Vector Laboratories, Burlingame, CA); rabbit anti-KRT5 (PA5-32465, Thermo-Fisher,), rabbit anti-ARL13B (17711-1-AP, Proteintech, Rosemont, IL), rabbit anti-SFTPA (HPA.42638, Sigma-Aldrich), rabbit anti-SFTPB (LS-B8080-50, Lifespan Bioscience, Seattle, WA), rabbit anti-FOXI1 (HPA071469, Sigma-Aldrich, St. Louis, MO), rabbit anti-MUC5B (HPA008246, Sigma-Aldrich, St. Louis, MO) and rabbit anti-MSMB (HPA051257, Sigma-Aldrich). The specificity of the primary antibodies has been tested on commercial human tissues (US Biomax, Rockville, MD). Secondary antibodies were Alexa 555 or 488 or 647-labeled antibodies (Thermo-Fisher). The nucleus was stained by DAPI (Thermo-Fisher). Images were captures using an Olympus IX71 inverted microscope with an Olympus DP73 camera. Exposures in fluorescence channels were merged using ImageJ (imagej.nih.gov).

### Karyotyping

Karyotype analysis of the immortalized hSABCi-NS1.1 cell at passage 50 was performed at the Molecular Cytogenetics-Core Facility at Memorial Sloan-Kettering Cancer Center using established protocols [21]. At least 20 metaphase spreads were analyzed. All metaphases were fully karyotyped.

### Real time PCR

cDNA was synthesized using TaqMan Reverse Transcriptase Reaction kit (Thermo-Fisher). All reactions were run on an Applied Biosystems Sequence Detection System 7500. Relative expression levels were determined using the △Ct method with 18S ribosomal RNA as the endogenous control. Premade TaqMan Gene Expression Assays (Thermo-Fisher) were used as listed in Additional file 1: Table S1.

### Microarray and RNAseq

Trachea, large airway epithelium (LAE, 3rd -4th order bronchi) and small airway epithelium (SAE, 10th to 12th order bronchi) were collected from normal individuals via flexible bronchoscopy as described previously [13, 17, 20, 23]. Following RNA extraction and sample quality assessment, genome-wide expression assessment was performed using microarray (Human Genome U133 Plus 2.0 arrays, Affymetrix, Santa Clara, CA). The raw microarray data of the 9 matched trachea, large and small airway epithelium, and the SAE from 36 COPD smokers and 60 healthy nonsmokers have been published (GSE64614, GSE76327). The cluster analysis of cell line donor’s SAE with the 9 matched trachea, large and small airway epithelium was performed in Partek Genomics Suite (Partek, St. Louis, MO). Genes differentially expressed between the 9 paired trachea and SAE (fold changes > 2 fold, Benjamini-Hochberg corrected *p* < 0.05) were used as filter to generate heat map. For RNA-Seq (Illumina flow cells, San Diego, CA) [18], samples of passage 47 hSABCi-NS1.1 cell at basal stage (right before ALI culture) and ALI-d28 were used. The data are publicly available in the NCBI Gene Expression Omnibus (GEO accession number: GSE124265).

### Lentivirus-mediated SPDEF expression

The methods for lentivirus production were as previously described [22, 23]. Lenti-SPDEF-IRES-GFP (expressing SAM Pointed Domain Containing ETS Transcription Factor) and lenti-IRES-GFP (control) plasmids were generated using the PLKO lentiviral vector backbone (Thermo Scientific). The replication deficient lentiviruses were generated in 293A cells using compatible packaging vectors. Both viruses were pseudo-typed with the VSV-G envelope and concentrated with PEG-it™ Virus Precipitation Solution (System Biosciences, Mountain View, CA) using the manufacturer’s protocol. For infection of the cells, recombinant lentiviruses were added to basal cells immediately prior to plating on Transwell inserts by combining lentiviruses with suspended basal cells at a multiplicity of infection sufficient to produce > 90% positive GFP staining. The cells and virus were deposited in the apical chamber of the transwell insert at a concentration of 1.5 × 10^5^ cells/100 μl/well (designated ALI-day − 2; i.e., two days before removal of the apical medium). The following day, the infectious medium was removed, and the ALI culturing protocol continued as described above. The infection efficiency was confirmed by monitoring GFP positive staining.

### Statistical analysis

The two-tailed Student’s t-test was used to compare gene expression in both in vivo and in vitro data. In all analyses, a *p* value less than 0.05 was deemed significant.

## Results

### Generation of hSABCi-NS1.1

**Fig. 1:**
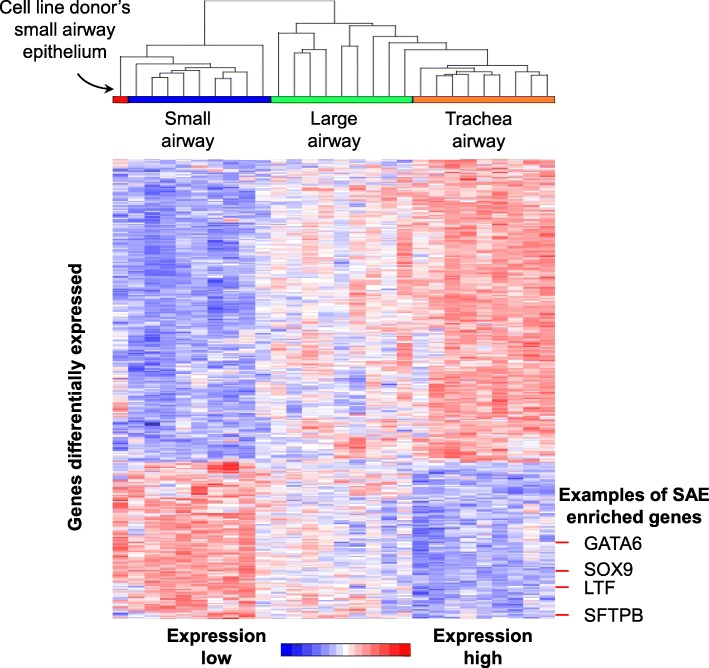
Typical small airway transcriptome features of the cell line donor’s small airway epithelium (SAE). Data shown is the unsupervised cluster analysis of microarray data from the cell line donor’s small airway epithelium with data from previously published-microarray datasets that include 9 matched-trachea, large airway and small airway epithelium samples. Genes differentially expressed between the paired trachea and SAE (fold changes > 2 fold, Benjamini-Hochberg corrected p < 0.05) were selected to generate the plot. Examples of SAE-enriched genes (GATA6, SOX9, LTF and SFTPB) are indicated. The donor’s SAE clusters with the reference SAE transcriptome, distinct from the large airway and trachea epithelium

Based on our previous published sub-dataset [20], small airway epithelium has a different gene expression pattern than matched-tracheal and large airway epithelium from healthy nonsmokers (Fig. 1). For example, expression of SFTPB (surfactant protein), LTF (secretory cell gene) and small airway development-associated transcription factors GATA6 and SOX9 [24–27] are enriched in the small airway epithelium (Fig. 1). To ensure that the small airway epithelium recovered from the donor had typical SAE transcriptome, unsupervised clustering was carried out on the SAE transcriptome of the donor to compare with the previous small, large and trachea epithelium dataset. As expected, the microarray data of the donor clustered with the SAE samples when differential expression gene list of trachea vs small was assessed.

**Fig. 2:**
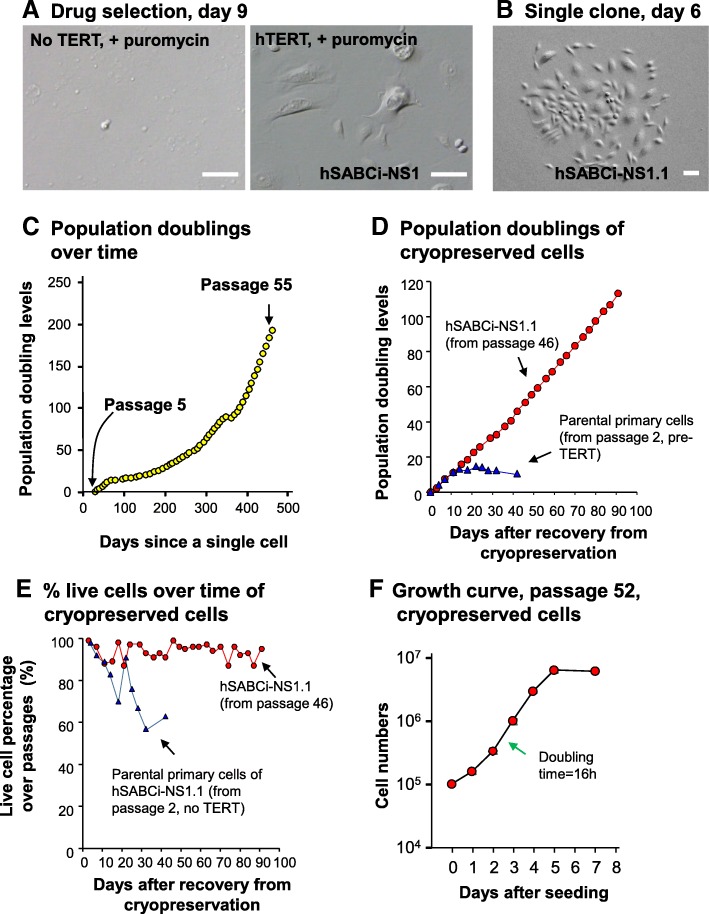
Isolation and growth of the hSABCi-NS1.1 immortalized small airway basal cells from a healthy nonsmoker subject. **a** Morphology of cells following drug selection, day 9. *Left* - parental cells without TERT, plus puromycin selection; *right* - hTERT infected cells with puromycin selection (mixed clones, hSABCi-NS1). Bar = 100 μm. **b** Morphology of the single cell-derived cell clone hSABCi-NS1.1 on day 6, passage 0. Single-cell clones were generated by culturing limited-numbers of cells of hSABCi-NS1 cell in 10 cm plate and tracked under the microscope from day 2. Bar = 100 μm. **c** Growth of hSABCi-NS1.1. Cell population doubling levels were quantified from passage 5. Each dot represents one passage in T25 flask. **d** Growth of hSABCi-NS1.1 after recovery from cryopreservation. Passage 46 of hSABCi-NS1.1 cells (red dots) which had been frozen in the liquid nitrogen for 131 days, was re-cultured to test the consistence of growth. The population doubling levels were re-counted from the day of re-culturing. Passage 1 parental pre-immortalized primary cells were cultured at the same time as control (blue triangles). **e** % live cells of the re-cultured cells. The % live cells of the re-cultured hSABCi-NS1.1 cells were counted during each passage. Only adherent cells were counted. Red – hSABCi-NS1.1, passage 46; blue – parental primary cells pre-TERT, passage 2. **f** Growth of the re-cultured hSABCi-NS1.1 cells at passage 52. Cell doubling time between day 2 and day 3 after seeding was 16 h. The rate of cell doubling was reduced after day 5 when the cells reached confluence

After retro-hTERT genetic modification, the SABC were resistant to puromycin selection (Fig. 2a). The resulting cell population was a mixed cell population termed as hSABCi-NS1. A single cell clone was isolated from hSABCi-NS1 (termed as hSABCi-NS1.1) (Fig. 2b). The heterogeneous morphology is likely because these cells were at different phases of the cell [22]. The hSABCi-NS1.1 clone survived and was expanded for more than 1 year in vitro (Fig. 2c)*.* The cells entered consistent growth after 200 days. The population doubling levels between passage 5 and passage 55 were ~ 200 (Fig. 2c). As additional quality control, one vial of passage 46 hSABCi-NS1.1 cells, frozen in liquid nitrogen for > 100 days, was re-cultured and compared with a vial of passage 1 parental primary cells under the same culture conditions at the same time. Even after > 110 additional population doubling levels, the re-cultured passage 46 hSABCi-NS1.1 cells had no signs of replicative senescence (Fig. 2d, Additional file 1: Figure S1). The percentage of live cells over each passage in the re-cultured cells was always > 80% (Fig. 2e). The doubling time of hSABCi-NS1.1 cells during exponential growth of passage 52 was approximately 16 h (Fig. 2f). Based on morphology, the cells remain healthy in the late passages.

Karyotyping analysis of 23 hSABCi-NS1.1 cells from passage 50 cell showed that all of the cells hade an abnormal male karyotype with trisomy 8 in all cells analyzed. Additionally, 22 (96%) cells contained abnormalities of chromosome 19 (resulting in net gain of 19q and/or 19p) and 15 (65%) cells contained trisomy 20.

### Basal/stem cell markers

**Fig. 3:**
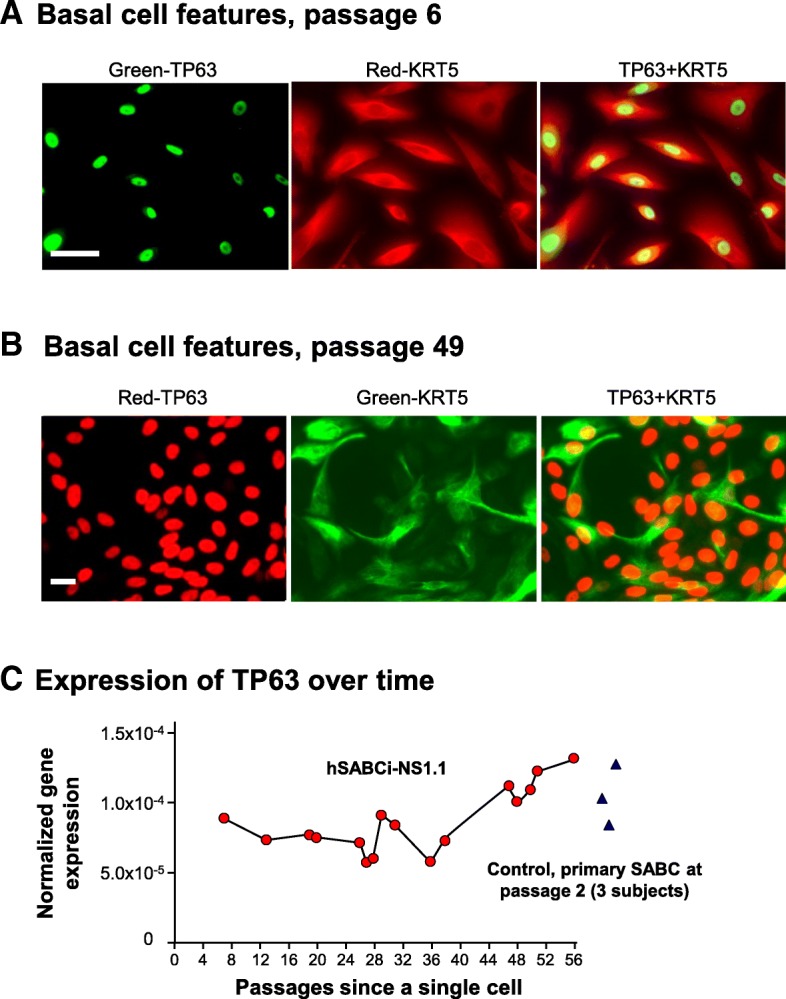
Maintenance of basal cell features in the immortalized hSABCi-NS1.1 cells. **a** Basal cell markers staining at passage 6. *Left*, TP63 (green); *middle*, KRT5 (red); *right*, merged image. Bar = 50 μm. **b** Basal cell marker staining at passage 49. *Left*, TP63 (red); *middle*, KRT5 (green); *right*, merged image. Bar = 20 μm. Note that some nuclei appear negative for TP63 because of image contrast. **c** Expression of TP63 mRNA. Gene expression was assessed by TaqMan PCR. Each circle is one passage of hSABCi-NS1.1 cells. Primary small airway basal cells from 3 healthy nonsmokers at passage 2 were used as controls (blue triangle on right)

Consistent with the source of the hSABCi-NS1.1 cell line, immunostaining showed that the parental cells prior to immortalization expressed KRT5 (Additional file 1: Figure S2). hSABCi-NS1.1 cells were positive for basal cells markers, TP63 and KRT5, at passage 6 (Fig. 3a). The positive staining was maintained at passage 49 (Fig. 3b). TaqMan PCR confirmed that hSABCi-NS1.1 (passage 8 to passage 54) had similar mRNA expression levels of TP63 compared with primary small airway basal cells (Fig. 3c).

### Differentiation capacity

**Fig. 4:**
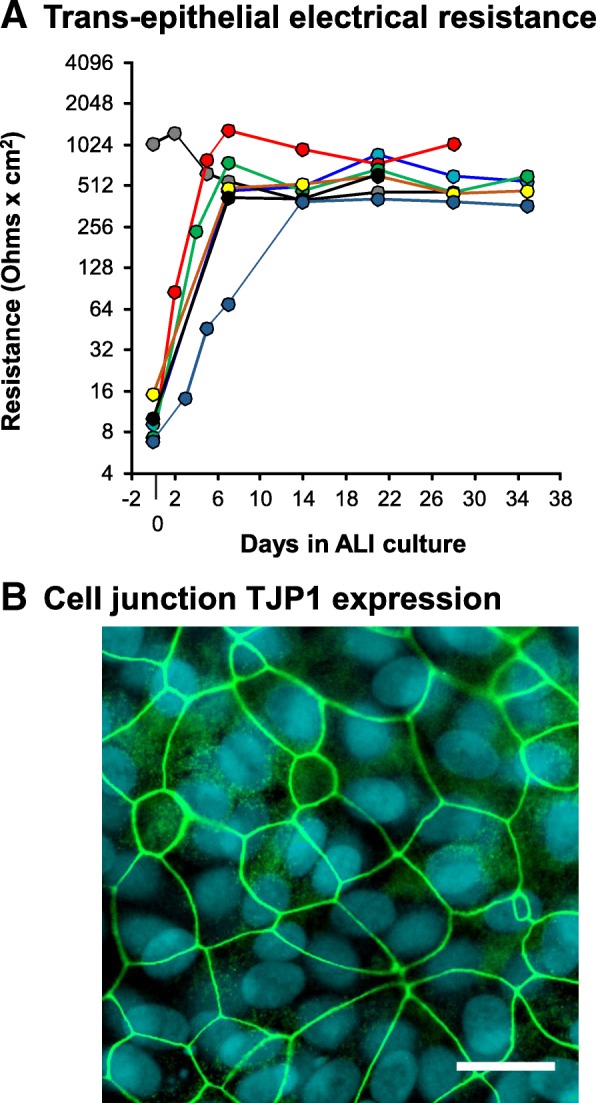
Cell junction formation of the immortalized hSABCi-NS1.1 cells on air-liquid interface (ALI) culture. **a** Trans-epithelial electrical resistance (TEER). Each color represents one independent experiment (*n* = 7 in total, passage 47 to 56 cells). Each dot represents average TEER of 3 ALI wells between day 0 and day 35 of ALI culture. **b** Junction protein staining of hSABCi-NS1.1 on ALI culture. Shown is tight junction protein 1 (TJP1) staining (green) of passage 47 cells on ALI-day 35. Bar = 20 μm. This composite image was created from a stack of individual images acquired at different depths in the monolayer (z-stack covering 40 μm). As a result, the merged image includes cell junction staining at the apical surface overlaid on the image of the epithelial cell nuclei. The difference in depth of the two structures accounts for difference in alignment of nuclei and apical tight junctions

To assess whether the hSABCi-NS1.1 cells still retain the potential to form tight junctions, the cells were cultured on ALI. Measurement of trans-epithelial electrical resistance showed that the resistances were consistently > 200 Ohms x cm^2^ after ALI-day 14 (Fig. 4a). Immunofluorescence staining revealed that there was sharp and continued tight junction protein 1 (TJP1) staining between the cells on the apical side (Fig. 4b).

**Fig. 5:**
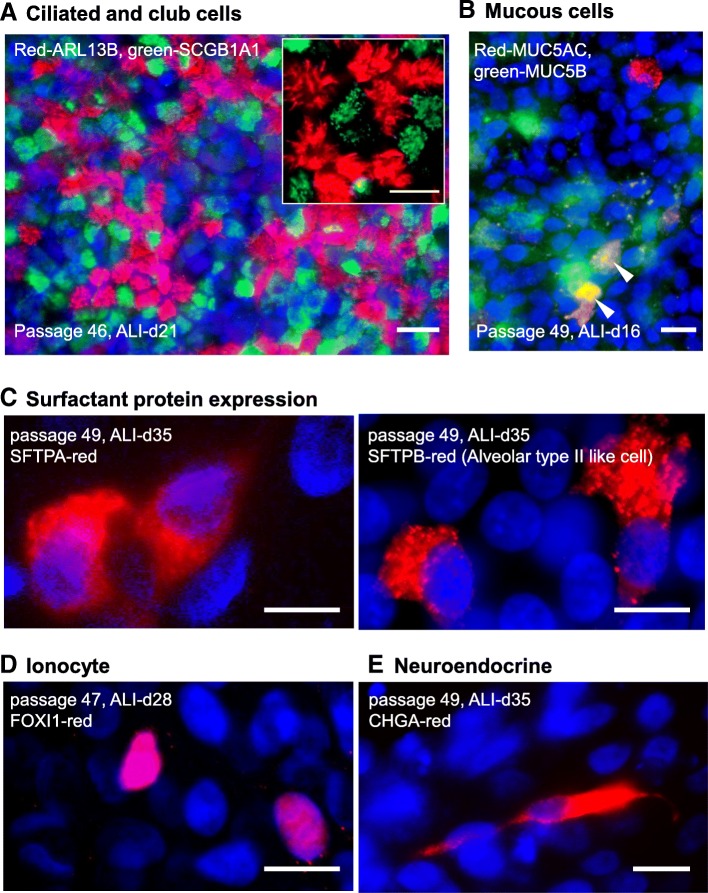
Differentiation capacity of immortalized hSABCi-NS1.1 cells on ALI culture. Passage 47 to passage 56 of hSABCi-NS1.1 cells were assessed on ALI day 14 to 35 for airway epithelial cell lineage markers. **a** Ciliated and club cells of passage 49 cells, ALI-day 21. Red-ARL13B; Green-SCGB1A1. Inset, higher magnification, showing cilia and SCGB1A1^+^ granules. Bar = 20 μm. **b** Mucous cells in passage 49 cells, ALI-day 16. Red-MUC5AC; green-MUC5B; yellow-co-localization (*arrowheads*). Bar = 20 μm. **c** Surfactant protein expressing cells, passage 49, ALI-day 35. *Left*, red-SFTPA; *right*, red-SFTPB. In both fields, nuclei were stained with DAPI (blue). **d** Ionocyte, passage 47, ALI-day 28. Red-FOXI1. Nuclei were stained with DAPI (blue). **e** Neuroendocrine cells, passage 49 cells, ALI-day 35. Red-CHGA. Nuclei were stained with DAPI (blue). Bar = 10 μm

To assess the differentiation capacity of hSABCi-NS1.1 cells on ALI, the ALI cells were stained with markers for SAE-associated lineages (Fig. 5a-e), including ciliated (ARL13B^+^ motile cilia), club (SCGB1A1^+^ organelles), mucous (MUC5AC^+^ or MUC5B^+^ organelles), ionocytes (FOXI1^+^ nuclei), neuroendocrine (CHGA^+^ organelles), and less-defined alveolar-like cells (SFTPA^+^ or SFTPB+ organelles). ARL13B staining showed a characteristic apical collection of curvilinear structures while SCGB1A1, MUC5AC, MUC5B, SFTPA, SFTPB, and CHGA staining occurred in collections of intracellular puncta typical of secretory vesicles. The transcription factor, FOXI1, co-localized with nuclei consistent with its role in regulation of gene expression. Ciliated cell and club cell staining were non-overlapping while MUC5AC and MUC5B were partially overlapping as expected.

RNAseq was used to confirm the differentiation capacities of hSABCi-NS1.1 on ALI. Compared to the undifferentiated basal cell stage, ALI-day 28 hSABCi-NS1.1 had down-regulation of basal cell genes (KRT5, KRT14, TP63, KRT15), with up-regulation of genes typical of ciliated cells (FOXJ1, DNAI1, SPAG6, TEKT1), secretory cells (SCGB1A1, SCGB3A1, MUC5B, LYZ), alveolar-like cells (SFTPA1, SFTPA2, SFTPB and SFTPD), neuroendocrine cells (CHGB and SCG5), ionocyte cells (FOXI1, ATP6V1B1 and CFTR; Table 1). All of these markers have been detected in small airway epithelium observed by brushing during fiberoptic bronchoscopy using RNAseq [17]. By functional term enrichment analysis, “cilium” and “immunity” were among the top 3 categories in the top 1500 up-regulated genes (Table 2), while “ribosomal protein” was the top 1 functional category associated with the top 1500 down-regulated genes (Table 2), consistent with the fact that basal cells contain abundant ribosomes [12]. In addition, many Notch pathway genes showed more than 1.5-fold changes during differentiation, e.g., NOTCH1 (− 2.1-fold), NOTCH2 (2.7-fold), NOTCH4 (1.5-fold), JAG2 (− 2.9-fold), HES1 (− 6.4-fold), HES2 (− 8.6-fold), HES4 (− 1.6-fold), HES6 (3.0-fold), HEY1 (1.8-fold) and HEYL (2.9-fold). The data is consistent with the knowledge that the dynamic changes of Notch pathway are involved in the commitment of airway epithelial lineages.

**Table 1:** Expression of airway epithelial lineage markers in hSABCi-NS1.1 cells during differentiation on ALI culture^a^

Cell lineages^b^	Gene symbol	Gene name	Expression level (FPKM)^c^	
			Basal	ALI-d28
Basal cells	KRT5	Keratin 5	130.9	121.2
	KRT14	Keratin 14	38.9	0.1
	TP63	Tumor protein p63	38.2	12.4
	KRT15	Keratin 15	1009.4	173.6
Ciliated cells	FOXJ1	Forkhead box J1	0.0	42.4
	DNAI1	Dynein axonemal intermediate chain 1	0.0	21.3
	SPAG6	Sperm associated antigen 6	0.0	7.6
	TEKT1	Tektin 1	0.1	40.5
Secretory cells	SCGB1A1	Secretoglobin family 1A member 1	35.6	6695.5
	SCGB3A1	Secretoglobin family 3A member 1	19.0	451.6
	MUC5B	Mucin 5B, Oligomeric mucus/gel-forming	2.0	22.3
	LYZ	Lysozyme	1.3	8.8
Alveolar cells	SFTPA1	Surfactant protein A1	0.0	0.1
	SFTPA2	Surfactant protein A2	0.0	1.0
	SFTPB	Surfactant protein B	0.0	3.6
	SFTPC	Surfactant protein C	0.0	0.0
	SFTPD	Surfactant protein D	0.1	0.5
Neuroendocrine cells	CHGA	Chromogranin A	0.0	0.0
	CHGB	Chromogranin B	0.8	1.2
	CALCA	Calcitonin-related polypeptide alpha	0.1	0.0
	SCG5	Secretogranin V	0.1	6.3
Ionocytes	FOXI1	Forkhead Box I1	0.0	0.1
	ATP6V1B1	ATPase H+ transporting V1 subunit B1	1.2	6.0
	CFTR	Cystic fibrosis transmembrane conductance regulator	0.0	11.4

^a^Expression level in hSABCi NS1.1 cells on ALI-day 28 compared to the basal cells prior to ALI culture were assessed by RNAseq

^b^Lineage markers were selected from the literature [12, 17, 24–27]

^c^FPKM - fragments per kilobase of transcript/million mapped reads

**Table 2:** Gene function annotation enrichment analysis of the top up-regulated and down-regulated genes during differentiation on ALI culture^a^

Term	Gene number^b^	Percentage of genes in the list (%)	*p* value^c^	*p* value after Benjamini-Hochberg correction
Top up-regulated				
Cilium	68	4.7	2.3 × 10^−28^	1.1 × 10^−25^
Cell projection	127	8.9	1.9 × 10^−23^	4.6 × 10^−21^
Immunity	101	7.0	3.4 × 10^−23^	5.3 × 10^− 21^
Top down-regulated				
Ribosomal protein	92	6.3	5.1 × 10^−57^	2.5 × 10^−54^
Ribonucleoprotein	102	6.9	4.0 × 10^−45^	9.6 × 10^−43^
Acetylation	380	25.9	2.6 × 10^−26^	4.1 × 10^−24^

^a^Function term annotation enrichment analysis was carried out using DAVID analysis (https://david.ncifcrf.gov/tools.jsp). The fold-changes of RNAseq data between hSABCi NS1.1 cells on ALI-day 28 was compared to basal cells before ALI culture. The top 1500 up-regulated genes and top 1500 down-regulated genes were used for analysis. The top 3 enriched functional terms in the results were shown. See Methods for detail

^b^Numbers of genes with the function described in “Term”

^c^*p* value is from a modified Fisher exact test, indicating whether the gene list is enriched with an annotated function or not

**Fig. 6:**
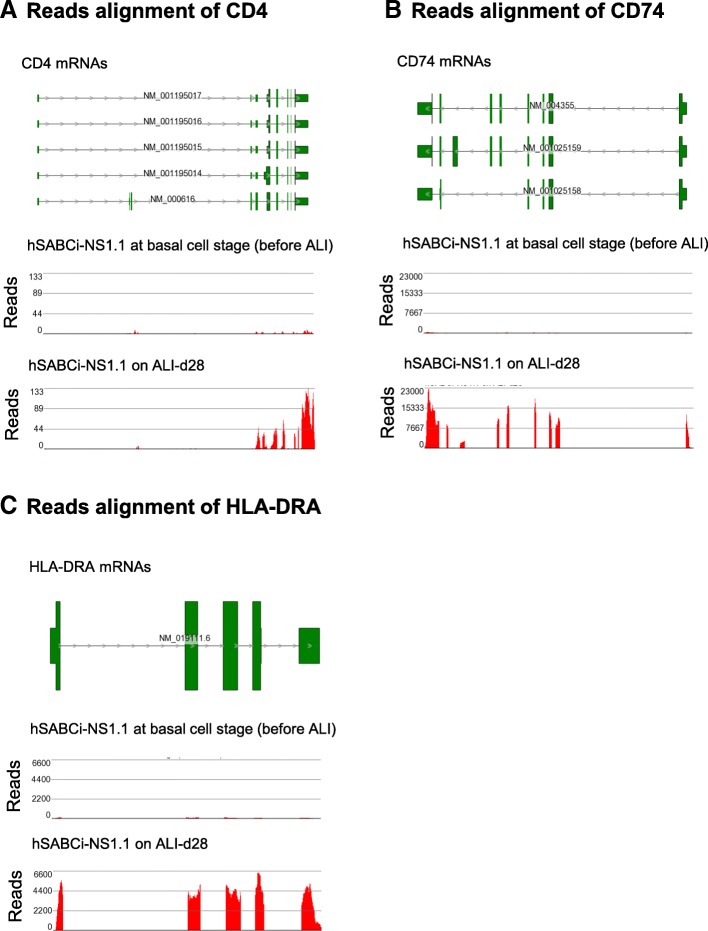
Example of non-inflammatory cell immunity genes in hSABCi-NS1.1 on ALI. The reads from the RNAseq data were aligned to the introns and exons of reference mRNAs of the selected genes. Both basal and ALI-d28 data were plotted. **a** CD4 alignment; **b** CD74 alignment; **c** HLA-DRA alignment

Interestingly, differentiated hSABCi-NS1.1 cells expressed inflammatory cell genes CD4 (lymphocyte cell marker) and MHC-II genes (CD74, HLA-DRA; dendritic cell markers; Additional file 1: Table S2). For additional confirmation, RNAseq reads were aligned with exons of selected mRNAs. The alignment clearly demonstrated the expression of immunity genes CD4, CD74 and HLA-DRA (Fig. 6a, b and c). Since hSABCi-NS1.1 was single-cell-derived, contamination with inflammatory cells can be excluded. The findings have corroborated our previous observation from single-cell RNAseq data that some airway epithelial cells were positive for inflammatory cell genes, including CD4, CD74 and HLA-DRA [12].

### Regional identity in the airways

**Fig. 7:**
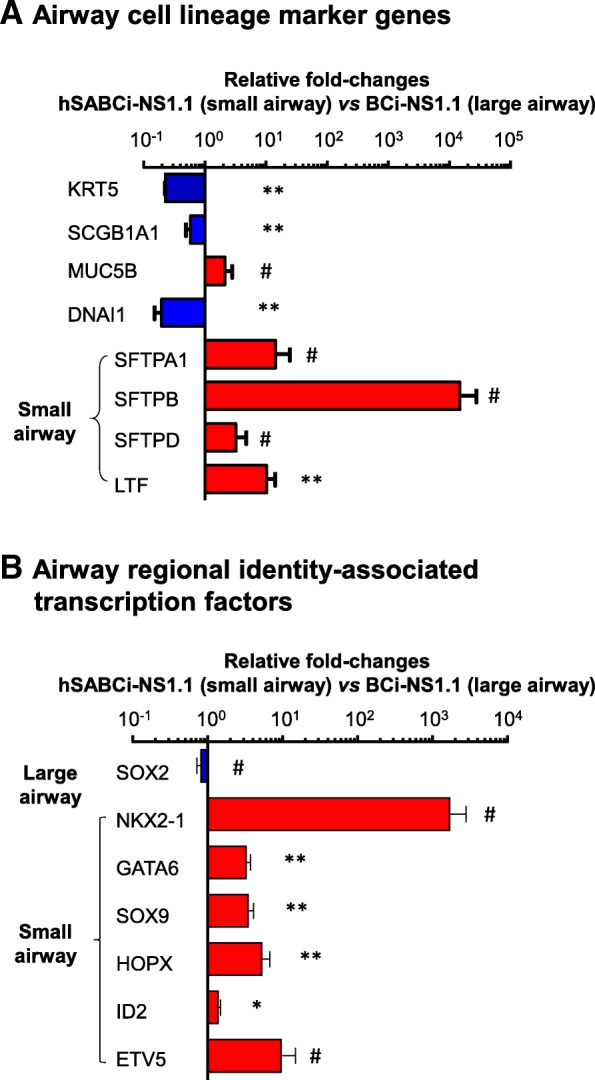
Expression of small airway development-relevant transcription factors in immortalized hSABCi-NS1.1 cells. Gene expression in hSABCi-NS1.1 cells (from small airway) was compared with gene expression in BCi-NS1.1 cells (from large airway). Both cell lines (passage 52–55) were cultured on ALI using identical culture conditions for comparison. Large and small airway lineage markers and transcription factors relevant to the development of large airway (SOX2) and small airway (NKX2–1, GATA6, SOX9, HOPX, ID2 and ETV5) were assessed by TaqMan PCR. Data shown is the mean fold-changes ± standard error of *n* = 3 independent side-by-side comparison experiments. *, *p* < 0.05; **, *p* < 0.01; #, consistent changes in all 3 independent experiments, but not significant because of high variability. **a** Airway cell lineage marker genes. **b** Transcription factors relevant to the development of large airway and small airway epithelium

To assess whether differentiation of airway cell lines have regionally enriched features, the transcriptome of hSBACi-NS1.1 cells was compared with our previously-reported large airway cell line BCi-NS1.1 [21] using the same culture condition in basal and ALI culture. TaqMan PCR analysis demonstrated that hSABCi-NS1.1 cells had lower expression levels of basal, club, and ciliated cell genes, while significantly higher expression levels of small airway-associated genes, including SFTPA1, SFTPB, SFTPD and LTF (Fig. 7a). Consistent with this data, there was a trend of lower expression of SOX2 (proximal airway transcription factor) and significantly higher expression levels of small airway development transcription factors (NXK2–1, GATA6, SOX9, HOPX, ID2, ETV5) [24–27] (Fig. 7b).

### Recapitulation of SAE-related disease

**Fig. 8:**
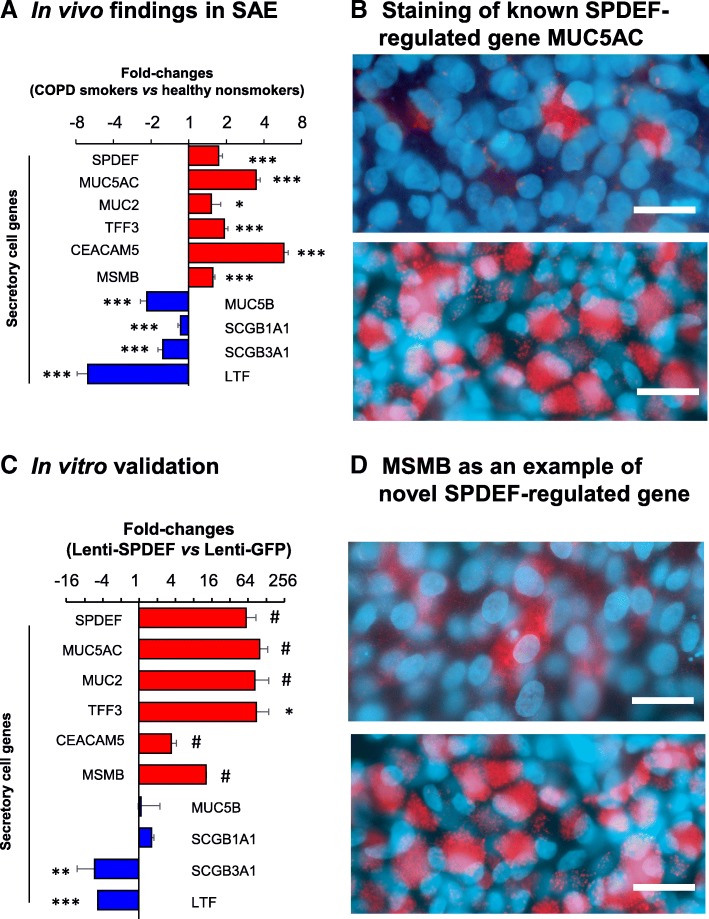
Consequences of overexpression of SPDEF on the differentiation of immortalized hSABCi-NS1.1 cells on ALI, as a model of secretory cell hyperplasia in COPD. Based on the knowledge that SPDEF, a transcription factor that induced secretory cell differentiation is up-regulated in the SAE of COPD smokers. hSABCi-NS1.1 cells were genetically modified by lentivirus expressing SPDEF and cultured on ALI to day 14 to test whether the hSABCi-NS1.1 cells can be used as a model of secretory cell hyperplasia. **a** Secretory cell gene expression in the SAE of 36 COPD smokers compared to 60 healthy nonsmokers in vivo. Five up-regulated and 4 down-regulated secretory cell genes were used as examples. Data shown is relative fold-change of microarray-based gene expression ± standard error. **b** Staining of a known SPDEF-regulated gene in ALI. MUC5AC was used as an example. Shown are passage 47 cells on ALI-day 21. Red-MUC5AC; blue-nucleus. Top panel, lenti-GFP infected cells, as control; bottom panel, lenti-SPDEF-infected cells. Bar = 50 μm. **c** TaqMan PCR assessment of lentivirus-SPDEF-induced gene expression changes in hSABCi-NS1.1 cells. The same genes as in panel A were assessed. Data shown is the mean fold-change ± standard error of n = 3 independent experiments using passage 52 to passage 55 cells. **d** Staining of MSMB as an example of a SPDEF-regulated secretory gene in ALI. Shown are passage 47 cells on ALI-day 21. Red-MSMB; blue-nucleus. Top panel, lenti-GFP infected cells, as control; bottom panel, lenti-SPDEF-infected cells. For **a**, **c** *, *p* < 0.05; **, *p* < 0.01; ***, *p* < 0.001; #, consistent changes in all 3 independent experiments, but not significant because of variability

To test potential applications of the cell line in the studies of SAE diseases, hSABCi-NS1.1 cells were genetically modified by lentivirus to express SPDEF, a key transcription factor controlling secretory cell differentiation [28]. As shown in Fig. 8a, SAE from COPD smokers demonstrated dysregulation of multiple secretory cell genes, including SPDEF. Based on GFP distribution, the infection efficiency of lentivirus in hSABCi-NS1.1 cells was high and maintained during ALI culture (not shown). As a control, SPDEF overexpression in hSABCi-NS1.1 cells on ALI induced remarkable up-regulation of MUC5AC staining (Fig. 8b). TaqMan PCR data demonstrated that upregulation of SPDEF led to increased-expression of MUC5AC, MUC2, TFF3, CEACAM5, MSMB and decreased-expression of SCGB3A1 and LTF. However, no consistent changes in SCGB1A1 or MUC5B were found (Fig. 8c), supporting our previous proposal that a transcription factor network might be involved [29]. Immunostaining confirmed the striking up-regulation of MSMB (Fig. 8d), which has not been identified as a SPDEF-regulated gene in previous studies [28].

## Discussion

We have generated and characterized a single-cell-derived normal human small airway basal cell line (hSABCi-NS1.1) with diverse differentiation capacities and regional features. This human SAE cell line can achieve high cell doubling levels and passage numbers. The hSABCi-NS1.1 cell line provided a novel tool to study SAE biology and pathophysiology in an airway region relevant manner. The availability of hSABCi-NS1.1 cells will permit studies of diseases for which the SAE plays a central role. It also can be used to assess the effect of specific mediators on the SAE. As an example, we found transcription factor SPDEF can induce human COPD-relevant changes in hSABCi-NS1.1 cells. Also relevant to small airway biology, hSABCi-NS1.1 can differentiate to ionocytes, a recently identified airway cell type [30, 31], surfactant protein positive cells [13, 17, 20, 32] and neuroendocrine cells [17]. Basal cells are likely the common progenitor for all these cell types, and hSABCi-NS1.1 cells will be a convenient tool to study these cell populations.

The hSABCi-NS1.1 cell line demonstrates regional (small airway epithelium) identity. Along the proximal to distal axis, the airway epithelium has different morphological features [4, 7, 20, 24–27]. As examples, compared to the tracheal epithelium, small airway epithelium has a thinner epithelial layer, is composed of basal, secretory and ciliated cells in different ratios, and has surfactant protein positive cells [13, 14, 32]. These airway regional features are apparent in the airway epithelium transcriptome, including SAE-specific surfactant protein genes and SAE enrichment of distal airway patterning-associated transcription factors NKX2–1, GATA6, SOX9 and ETV5 [24–27].

The regional identity is likely directly involved in the pathogenesis of SAE-related diseases. For example, SAE express surfactant protein genes (SFTPB, SFTPA2), genetic variation of which are associated with COPD and IPF [33, 34]. SAE-enriched transcription factor, NKX2–1, is relevant to lung adenocarcinoma [35], a cancer type that is derived from the small airway epithelium. The expression of MUC5B, an IPF risk gene [36], is enriched in SAE [37]. GATA6 is involved in regulating MUC5AC gene expression [38] and can regulate an IPF-associated mesenchymal phenotype [39]. LTF plays important anti-infection roles in CF [40].

## Conclusion

In vitro differentiation models of airway epithelial cells have been widely used in studies for airway development, carcinogenesis, environmental insults and infection by pathogens [41, 42]. Importantly, since the SAE plays a central role in many chronic lung disorders, hSABCi-NS1.1 cells should be a valuable tool for drug screening. In this context, hSABCi-NS1.1 cells are available for other investigators by contacting the senior author.

## Additional file

Additional file 1: **Table S1.** List of primers for TaqMan assays. **Table S2.** List of immunity-related genes among the top 1500 up-regulated genes expressed by hSABCi-NS1.1 on air-liquid interface. **Figure S1.** Morphology of hSABCi-NS1.1 at late passage. **Figure S2.** Basal cell marker KRT5 staining in the parental cell prior to immortalization. (PDF 641 kb)

## Data Availability

The data are publically available in the NCBI Gene Expression Omnibus (GEO accession number: GSE124265).

## Abbreviations

ALI: Air-liquid interface
BC: Basal cells
COPD: Chronic obstructive pulmonary disease
IPF: Idiopathic pulmonary fibrosis
SABC: Small airway basal cell
SAE: Small airway epithelium
SAGM: Small airway epithelial cell growth medium
TEER: Transepithelial electrical resistance
TERT: Telomerase reverse transcriptase

## References

[1] Fulmer JD, Roberts WC, von Gal ER, Grystal RG (1977). Small airways in idiopathic pulmonary fibrosis. Comparison of morphologic and physiologic observations. J Clin Invest.

[2] Hogg JC, Chu F, Utokaparch S, Woods R, Elliott WM, Buzatu L, Cherniack RM, Rogers RM, Sciurba FC, Coxson HO, Pare PD (2004). The nature of small-airway obstruction in chronic obstructive pulmonary disease. N Engl J Med.

[3] Blouquit S, Regnier A, Dannhoffer L, Fermanian C, Naline E, Boucher R, Chinet T (2006). Ion and fluid transport properties of small airways in cystic fibrosis. Am J Respir Crit Care Med.

[4] Crystal RG, Randell SH, Engelhardt JF, Voynow J, Sunday ME (2008). Airway epithelial cells: current concepts and challenges. Proc Am Thorac Soc.

[5] McDonough JE, Yuan R, Suzuki M, Seyednejad N, Elliott WM, Sanchez PG, Wright AC, Gefter WB, Litzky L, Coxson HO (2011). Small-airway obstruction and emphysema in chronic obstructive pulmonary disease. N Engl J Med.

[6] Burgel PR, Bergeron A, de Blic J, Bonniaud P, Bourdin A, Chanez P, Chinet T, Dalphin JC, Devillier P, Deschildre A (2013). Small airways diseases, excluding asthma and COPD: an overview. Eur Respir Rev.

[7] Rock JR, Randell SH, Hogan BL (2010). Airway basal stem cells: a perspective on their roles in epithelial homeostasis and remodeling. Dis Model Mech.

[8] Crystal RG (2014). Airway basal cells. The "smoking gun" of chronic obstructive pulmonary disease. Am J Respir Crit Care Med.

[9] Staudt MR, Buro-Auriemma LJ, Walters MS, Salit J, Vincent T, Shaykhiev R, Mezey JG, Tilley AE, Kaner RJ, Ho MW, Crystal RG (2014). Airway basal stem/progenitor cells have diminished capacity to regenerate airway epithelium in chronic obstructive pulmonary disease. Am J Respir Crit Care Med.

[10] Xi Y, Kim T, Brumwell AN, Driver IH, Wei Y, Tan V, Jackson JR, Xu J, Lee DK, Gotts JE (2017). Local lung hypoxia determines epithelial fate decisions during alveolar regeneration. Nat Cell Biol.

[11] Yang Y, Riccio P, Schotsaert M, Mori M, Lu J, Lee DK, Garcia-Sastre A, Xu J, Cardoso WV (2018). Spatial-temporal lineage restrictions of embryonic p63(+) progenitors establish distinct stem cell pools in adult airways. Dev Cell.

[12] Zuo WL, Shenoy SA, Li S, O'Beirne SL, Strulovici-Barel Y, Leopold PL, Wang G, Staudt MR, Walters MS, Mason C (2018). Ontogeny and biology of human small airway epithelial Club cells. Am J Respir Crit Care Med.

[13] Harvey BG, Heguy A, Leopold PL, Carolan BJ, Ferris B, Crystal RG (2007). Modification of gene expression of the small airway epithelium in response to cigarette smoking. J Mol Med (Berl).

[14] Hackett NR, Shaykhiev R, Walters MS, Wang R, Zwick RK, Ferris B, Witover B, Salit J, Crystal RG (2011). The human airway epithelial basal cell transcriptome. PLoS One.

[15] Buro-Auriemma LJ, Salit J, Hackett NR, Walters MS, Strulovici-Barel Y, Staudt MR, Fuller J, Mahmoud M, Stevenson CS, Hilton H (2013). Cigarette smoking induces small airway epithelial epigenetic changes with corresponding modulation of gene expression. Hum Mol Genet.

[16] Deeb RS, Walters MS, Strulovici-Barel Y, Chen Q, Gross SS, Crystal RG (2016). Smoking-associated disordering of the airway basal stem/progenitor cell Metabotype. Am J Respir Cell Mol Biol.

[17] Hackett NR, Butler MW, Shaykhiev R, Salit J, Omberg L, Rodriguez-Flores JL, Mezey JG, Strulovici-Barel Y, Wang G, Didon L, Crystal RG (2012). RNA-Seq quantification of the human small airway epithelium transcriptome. BMC Genomics.

[18] Ryan DM, Vincent TL, Salit J, Walters MS, Agosto-Perez F, Shaykhiev R, Strulovici-Barel Y, Downey RJ, Buro-Auriemma LJ, Staudt MR (2014). Smoking dysregulates the human airway basal cell transcriptome at COPD risk locus 19q13.2. PLoS One.

[19] Wang G, Wang R, Strulovici-Barel Y, Salit J, Staudt MR, Ahmed J, Tilley AE, Yee-Levin J, Hollmann C, Harvey BG (2015). Persistence of smoking-induced dysregulation of miRNA expression in the small airway epithelium despite smoking cessation. PLoS One.

[20] Yang J, Zuo WL, Fukui T, Chao I, Gomi K, Lee B, Staudt MR, Kaner RJ, Strulovici-Barel Y, Salit J (2017). Smoking-dependent distal-to-proximal Repatterning of the adult human small airway epithelium. Am J Respir Crit Care Med.

[21] Walters MS, Gomi K, Ashbridge B, Moore MA, Arbelaez V, Heldrich J, Ding BS, Rafii S, Staudt MR, Crystal RG (2013). Generation of a human airway epithelium derived basal cell line with multipotent differentiation capacity. Respir Res.

[22] Zhou H, Brekman A, Zuo WL, Ou X, Shaykhiev R, Agosto-Perez FJ, Wang R, Walters MS, Salit J, Strulovici-Barel Y (2016). POU2AF1 functions in the human airway epithelium to regulate expression of host defense genes. J Immunol.

[23] Wang G, Zhou H, Strulovici-Barel Y, Al-Hijji M, Ou X, Salit J, Walters MS, Staudt MR, Kaner RJ, Crystal RG (2017). Role of OSGIN1 in mediating smoking-induced autophagy in the human airway epithelium. Autophagy.

[24] Maeda Y, Dave V, Whitsett JA (2007). Transcriptional control of lung morphogenesis. Physiol Rev.

[25] Morrisey EE, Hogan BL (2010). Preparing for the first breath: genetic and cellular mechanisms in lung development. Dev Cell.

[26] Herriges M, Morrisey EE (2014). Lung development: orchestrating the generation and regeneration of a complex organ. Development.

[27] Nikolić Marko Z., Sun Dawei, Rawlins Emma L. (2018). Human lung development: recent progress and new challenges. Development.

[28] Chen G, Korfhagen TR, Xu Y, Kitzmiller J, Wert SE, Maeda Y, Gregorieff A, Clevers H, Whitsett JA (2009). SPDEF is required for mouse pulmonary goblet cell differentiation and regulates a network of genes associated with mucus production. J Clin Invest.

[29] Wang G, Xu Z, Wang R, Al-Hijji M, Salit J, Strulovici-Barel Y, Tilley AE, Mezey JG, Crystal RG (2012). Genes associated with MUC5AC expression in small airway epithelium of human smokers and non-smokers. BMC Med Genet.

[30] Montoro DT, Haber AL, Biton M, Vinarsky V, Lin B, Birket SE, Yuan F, Chen S, Leung HM, Villoria J (2018). A revised airway epithelial hierarchy includes CFTR-expressing ionocytes. Nature.

[31] Plasschaert LW, Zilionis R, Choo-Wing R, Savova V, Knehr J, Roma G, Klein AM, Jaffe AB (2018). A single-cell atlas of the airway epithelium reveals the CFTR-rich pulmonary ionocyte. Nature.

[32] Griese M (1999). Pulmonary surfactant in health and human lung diseases: state of the art. Eur Respir J.

[33] Foreman MG, DeMeo DL, Hersh CP, Carey VJ, Fan VS, Reilly JJ, Shapiro SD, Silverman EK (2008). Polymorphic variation in surfactant protein B is associated with COPD exacerbations. Eur Respir J.

[34] Evans CM, Fingerlin TE, Schwarz MI, Lynch D, Kurche J, Warg L, Yang IV, Schwartz DA (2016). Idiopathic pulmonary fibrosis: a genetic disease that involves Mucociliary dysfunction of the peripheral airways. Physiol Rev.

[35] Tanaka H, Yanagisawa K, Shinjo K, Taguchi A, Maeno K, Tomida S, Shimada Y, Osada H, Kosaka T, Matsubara H (2007). Lineage-specific dependency of lung adenocarcinomas on the lung development regulator TTF-1. Cancer Res.

[36] Seibold MA, Wise AL, Speer MC, Steele MP, Brown KK, Loyd JE, Fingerlin TE, Zhang W, Gudmundsson G, Groshong SD (2011). A common MUC5B promoter polymorphism and pulmonary fibrosis. N Engl J Med.

[37] Okuda Kenichi, Chen Gang, Subramani Durai B., Wolf Monroe, Gilmore Rodney C., Kato Takafumi, Radicioni Giorgia, Kesimer Mehmet, Chua Michael, Dang Hong, Livraghi-Butrico Alessandra, Ehre Camille, Doerschuk Claire M., Randell Scott H., Matsui Hirotoshi, Nagase Takahide, O’Neal Wanda K., Boucher Richard C. (2019). Localization of Secretory Mucins MUC5AC and MUC5B in Normal/Healthy Human Airways. American Journal of Respiratory and Critical Care Medicine.

[38] Jonckheere N, Velghe A, Ducourouble MP, Copin MC, Renes IB, Van Seuningen I (2011). The mouse Muc5b mucin gene is transcriptionally regulated by thyroid transcription factor-1 (TTF-1) and GATA-6 transcription factors. FEBS J.

[39] Lepparanta O, Pulkkinen V, Koli K, Vahatalo R, Salmenkivi K, Kinnula VL, Heikinheimo M, Myllarniemi M (2010). Transcription factor GATA-6 is expressed in quiescent myofibroblasts in idiopathic pulmonary fibrosis. Am J Respir Cell Mol Biol.

[40] Rogan MP, Taggart CC, Greene CM, Murphy PG, O'Neill SJ, McElvaney NG (2004). Loss of microbicidal activity and increased formation of biofilm due to decreased lactoferrin activity in patients with cystic fibrosis. J Infect Dis.

[41] Gazdar AF, Girard L, Lockwood WW, Lam WL, Minna JD (2010). Lung cancer cell lines as tools for biomedical discovery and research. J Natl Cancer Inst.

[42] Miller AJ, Spence JR (2017). In vitro models to study human lung development, disease and homeostasis. Physiology (Bethesda).

