# Supplementary material for: Characterization of an immortalized human small airway basal stem/progenitor cell line with airway region-specific differentiation capacity
Source: Respir Res. 2019 Aug 23;20:196. doi: 10.1186/s12931-019-1140-9 (PMC6708250; doi:10.1186/s12931-019-1140-9)

Supplemental Table I. List of Primers for TaqMan Assays

Gene Symbol	Assay ID
CEACAM5	Hs00944025_m1
DNAI1	Hs00201755_m1
ETV5	Hs00927557_m1
GATA6	Hs00232018_m1
HOPX	Hs05028646_s1
ID2	Hs04187239_m1
KRT5	Hs00361185_m1
LTF	Hs00914334_m1
MUC2	Hs03005103_g1
MUC5AC	Hs01365616_m1
MUC5B	Hs00861595_m1
NKX2-1	Hs00968940_m1
SCGB1A1	Hs00171092_m1
SCGB3A1	Hs00369360_g1
SFTPA1	Hs00831305_s1
SFTPB	Hs00167036_m1
SFTPD	Hs01108490_m1
SOX2	Hs01053049_s1
SOX9	Hs00165814_m1
SPDEF	Hs00171942_m1
TFF3	Hs00902278_m1
TP63	Hs00978340_m1

Supplemental Table II. List of Immunity-related Genes Among the Top 1500 Up-regulated Genes Expressed by hSABCi-NS1.1 on Air-liquid Interface[1,2,3,4]

Gene symbol	Gene name	Expression level (FPKM)		Fold (ALI-d28/basal)
		Basal	ALI-d28	
BPIFB1	BPI fold containing family B member 1(BPIFB1)	17.7	2211.9	118.3
IDO1	indoleamine 2,3-dioxygenase 1(IDO1)	2.2	347.0	108.5
LTF	lactotransferrin(LTF)	0.0	75.6	76.6
IFITM1	interferon induced transmembrane protein 1(IFITM1)	7.5	499.6	58.9
IFIT3	interferon induced protein with tetratricopeptide repeats 3(IFIT3)	1.0	112.3	55.6
BST2	bone marrow stromal cell antigen 2(BST2)	6.8	402.9	52.0
HLA-DPA1	major histocompatibility complex, class II, DP alpha 1(HLA-DPA1)	1.9	126.4	44.1
C4BPA	complement component 4 binding protein alpha(C4BPA)	0.1	45.3	44.0
MX1	MX dynamin like GTPase 1(MX1)	2.6	148.9	42.1
CD74	CD74 molecule(CD74)	27.3	1185.0	42.0
HLA-DQA1	major histocompatibility complex, class II, DQ alpha 1(HLA-DQA1)	0.8	71.2	39.9
IFIT2	interferon induced protein with tetratricopeptide repeats 2(IFIT2)	0.6	56.3	36.4
HLA-DRA	major histocompatibility complex, class II, DR alpha(HLA-DRA)	12.2	476.7	36.3
IFIT1	interferon induced protein with tetratricopeptide repeats 1(IFIT1)	0.3	43.3	33.2
HLA-DQA2	major histocompatibility complex, class II, DQ alpha 2(HLA-DQA2)	0.4	46.0	33.1
HLA-DRB1	major histocompatibility complex, class II, DR beta 1(HLA-DRB1)	6.2	219.7	30.6
HP	haptoglobin(HP)	0.2	32.7	29.2
C4A	complement C4A (Rodgers blood group)(C4A)	0.1	30.5	28.5
CFI	complement factor I(CFI)	0.2	30.1	26.2
C1S	complement C1s(C1S)	0.6	34.9	22.6
MX2	MX dynamin like GTPase 2(MX2)	0.5	32.4	22.3
HLA-DMB	major histocompatibility complex, class II, DM beta(HLA-DMB)	1.1	43.7	21.2
TLR2	toll like receptor 2(TLR2)	1.0	40.2	21.0
SERPING1	serpin family G member 1(SERPING1)	1.2	44.0	20.6
BPIFA1	BPI fold containing family A member 1(BPIFA1)	72.5	1479.9	20.2
SAMHD1	SAM and HD domain containing deoxynucleoside triphosphate triphosphohy-	2.3	63.1	19.2

Supplemental Table II. List of Immunity-related Genes Among the Top 1500 Up-regulated Genes Expressed by hSABCi-NS1.1 on Air-liquid Interface[1,2,3,4] (cont., page 2)

Gene symbol	Gene name	Expression level (FPKM)		Fold (ALI-d28/basal)
		Basal	ALI-d28	
	drolase 1 (SAMHD1)			
RSAD2	radical S-adenosyl methionine domain containing 2 (RSAD2)	0.2	19.5	17.4
HLA-DQB1	major histocompatibility complex, class II, DQ beta 1 (HLA-DQB1)	0.8	29.1	17.1
HLA-DRB5	major histocompatibility complex, class II, DR beta 5 (HLA-DRB5)	1.6	39.7	15.6
HLA-DPB1	major histocompatibility complex, class II, DP beta 1(HLA-DPB1)	0.4	20.2	15.4
C1R	complement C1r (C1R)	2.9	50.1	13.1
VTCN1	V-set domain containing T cell activation inhibitor 1 (VTCN1)	1.0	24.4	12.5
HLA-DMA	major histocompatibility complex, class II, DM alpha (HLA-DMA)	7.4	101.1	12.2
C4B	complement C4B (Chido blood group) (C4B)	0.0	11.7	12.1
SUSD4	sushi domain containing 4 (SUSD4)	1.5	27.2	11.4
HLA-B	major histocompatibility complex, class I, B (HLA-B)	95.6	1081.1	11.2
DDX60	DExD/H-box helicase 60(DDX60)	1.6	25.8	10.4
TLR4	toll like receptor 4 (TLR4)	0.3	11.9	10.2
HERC5	HECT and RLD domain containing E3 ubiquitin protein ligase 5 (HERC5)	0.1	9.9	10.2
C6	complement C6 (C6)	0.0	9.0	10.0
TLR5	toll like receptor 5 (TLR5)	0.5	13.4	9.7
IFIH1	interferon induced with helicase C domain 1(IFIH1)	1.8	25.3	9.5
OASL	2'-5'-oligoadenylate synthetase like (OASL)	4.5	50.2	9.3
FGB	fibrinogen beta chain(FGB)	0.1	9.0	9.0
CLEC7A	C-type lectin domain family 7 member A (CLEC7A)	0.2	8.8	8.5
CSF1	colony stimulating factor 1 (CSF1)	4.2	40.4	8.0
HLA-DQB2	major histocompatibility complex, class II, DQ beta 2 (HLA-DQB2)	0.0	6.7	7.5
DDX58	DExD/H-box helicase 58 (DDX58)	2.9	28.3	7.4
NR1H4	nuclear receptor subfamily 1 group H member 4 (NR1H4)	0.0	6.4	7.4
GBP1	guanylate binding protein 1(GBP1)	5.9	49.9	7.4
HLA-F	major histocompatibility complex, class I, F (HLA-F)	8.8	69.4	7.2
LAMP3	lysosomal associated membrane protein 3 (LAMP3)	0.4	8.7	6.9
ISG15	ISG15 ubiquitin-like modifier (ISG15)	23.9	164.8	6.7

Supplemental Table II. List of Immunity-related Genes Among the Top 1500 Up-regulated Genes Expressed by hSABCi-NS1.1 on Air-liquid Interface[1,2,3,4] (cont., page 3)

Gene symbol	Gene name	Expression level (FPKM)		Fold (ALI-d28/basal)
		Basal	ALI-d28	
LBP	lipopolysaccharide binding protein (LBP)	0.0	5.6	6.3
OAS2	2'-5'-oligoadenylate synthetase 2 (OAS2)	12.3	82.9	6.3
LRMP	lymphoid restricted membrane protein (LRMP)	0.8	9.5	5.9
BTN3A1	butyrophilin subfamily 3 member A1(BTN3A1)	2.3	17.7	5.7
HLA-DOA	major histocompatibility complex, class II, DO alpha (HLA-DOA)	0.0	4.9	5.6
PSMB9	proteasome subunit beta 9 (PSMB9)	5.5	35.6	5.6
BTN3A2	butyrophilin subfamily 3 member A2(BTN3A2)	3.8	25.4	5.5
B2M	beta-2-microglobulin(B2M)	600.6	3191.9	5.3
CFB	complement factor B(CFB)	157.3	824.1	5.2
C2	complement C2(C2)	1.6	12.3	5.0
CD4	CD4 molecule (CD4)	0.3	5.4	4.9
SLFN11	schlafen family member 11(SLFN11)	0.3	5.0	4.7
HLA-C	major histocompatibility complex, class I, C (HLA-C)	182.0	857.2	4.7
NLRC5	NLR family CARD domain containing 5 (NLRC5)	0.2	4.5	4.6
BTN3A3	butyrophilin subfamily 3 member A3 (BTN3A3)	2.5	15.0	4.5
IFITM3	interferon induced transmembrane pro-tein 3 (IFITM3)	307.4	1386.4	4.5
OAS1	2'-5'-oligoadenylate synthetase 1 (OAS1)	17.0	77.9	4.4
IFIT5	interferon induced protein with tetratri-copeptide repeats 5 (IFIT5)	1.3	9.1	4.4
FGA	fibrinogen alpha chain (FGA)	0.0	3.3	4.2
GBP5	guanylate binding protein 5 (GBP5)	0.6	5.7	4.2
DHX58	DExH-box helicase 58 (DHX58)	1.9	11.0	4.2
THEMIS2	thymocyte selection associated family member 2 (THEMIS2)	1.5	9.2	4.0
OAS3	2'-5'-oligoadenylate synthetase 3(OAS3)	9.4	40.8	4.0
TLR3	toll like receptor 3 (TLR3)	0.9	6.2	3.9
IFITM2	interferon induced transmembrane pro-tein 2 (IFITM2)	32.4	128.1	3.9
ZC3HAV1	zinc finger CCCH-type containing, anti-viral 1(ZC3HAV1)	6.1	25.7	3.8
IRF1	interferon regulatory factor 1(IRF1)	6.9	28.7	3.8
SSC5D	scavenger receptor cysteine rich family member with 5 domains (SSC5D)	0.0	2.7	3.6
BCL6	B-cell CLL/lymphoma 6 (BCL6)	16.3	61.9	3.6
SMPDL3B	sphingomyelin phosphodiesterase acid like 3B(SMPDL3B)	0.9	5.8	3.6

Supplemental Table II. List of Immunity-related Genes Among the Top 1500 Up-regulated Genes Expressed by hSABCi-NS1.1 on Air-liquid Interface[1,2,3,4] (cont., page 4)

Gene symbol	Gene name	Expression level (FPKM)		Fold (ALI-d28/basal)
		Basal	ALI-d28	
SLAMF7	SLAM family member 7 (SLAMF7)	0.6	4.7	3.6
EIF2AK2	eukaryotic translation initiation factor 2 alpha kinase 2 (EIF2AK2)	2.8	12.1	3.5
LGALS9	galectin 9 (LGALS9)	21.9	75.4	3.3
SLPI	secretory leukocyte peptidase inhibitor (SLPI)	1612.0	5360.3	3.3
GATA3	GATA binding protein 3 (GATA3)	1.5	6.8	3.2
CFP	complement factor properdin (CFP)	0.0	2.3	3.1
IFI30	IFI30, lysosomal thiol reductase (IFI30)	28.1	90.0	3.1
CFH	complement factor H (CFH)	24.6	77.4	3.1
CD40	CD40 molecule (CD40)	1.9	7.8	3.0
TNFSF13	tumor necrosis factor superfamily member 13 (TNFSF13)	4.2	14.7	3.0
F2RL1	F2R like trypsin receptor 1 (F2RL1)	1.2	5.3	2.9
IL31RA	interleukin 31 receptor A (IL31RA)	0.2	2.4	2.9
TLR1	toll like receptor 1 (TLR1)	0.1	2.1	2.9
PML	promyelocytic leukemia (PML)	12.8	38.6	2.9
C4BPB	complement component 4 binding protein beta (C4BPB)	0.0	1.9	2.9
GBP3	guanylate binding protein 3 (GBP3)	19.2	56.5	2.9
ERAP1	endoplasmic reticulum aminopeptidase 1(ERAP1)	5.4	17.2	2.8
APOBEC3G	apolipoprotein B mRNA editing enzyme catalytic subunit 3G (APOBEC3G)	2.3	8.1	2.8

[1] The gene list with immunity functions were selected by DAVID Functional Annotation Bioinformatics Microarray Analysis (https://david.ncifcrf.gov/tools.jsp)

[2] Expression level of hSABCi NS1.1 on ALI-day 28 and basal cell stage before ALI culture were assessed by RNAseq.

[3] To avoid "0" in the denominators, the fold changes were based on "expression level + 1" of hSABCi NS1.1 at basal and ALI-d28 stage.

[4] The genes were ranked by fold-changes.

Supplemental Figure Legends

Supplemental Figure 1. In contrast to primary basal cells, immortalized hSABCi-NS1.1 cells do not demonstrate signs of cellular senescence after long-term culture. **A.** Morphology of the immortalized hSABCi-NS1.1 cell at passage 60. **B.** Morphology of the primary parental cells at passage 7. Red arrow, examples of cellular senescence.

Supplemental Figure 2. Basal cell marker KRT5 staining in the parental cell prior to immortalization. Green-KRT5; blue-nuclei. Bar=50 μm. **A.** KRT5 staining only; **B.** Color combine of KRT5 and nucleus staining.

A. Immortalized hSABCi-NS1.1 cells at passage 60

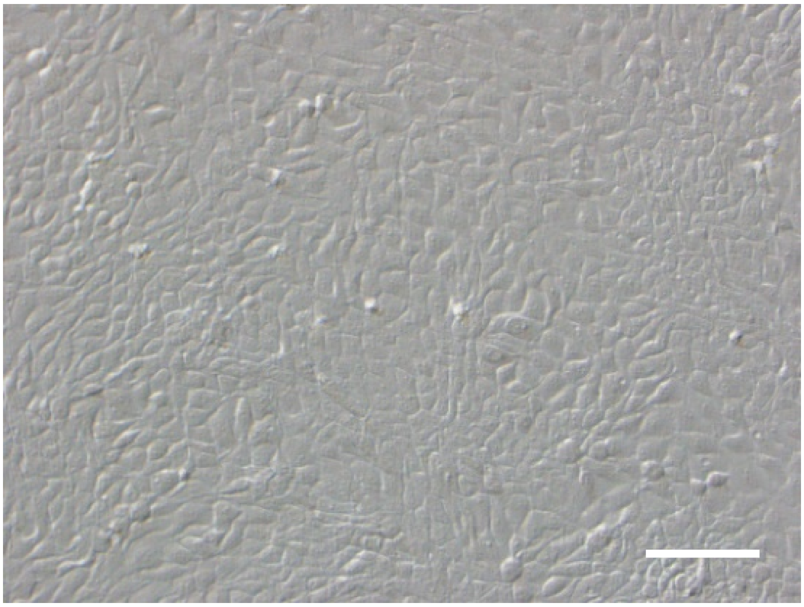

B. Primary parental cells at passage 7

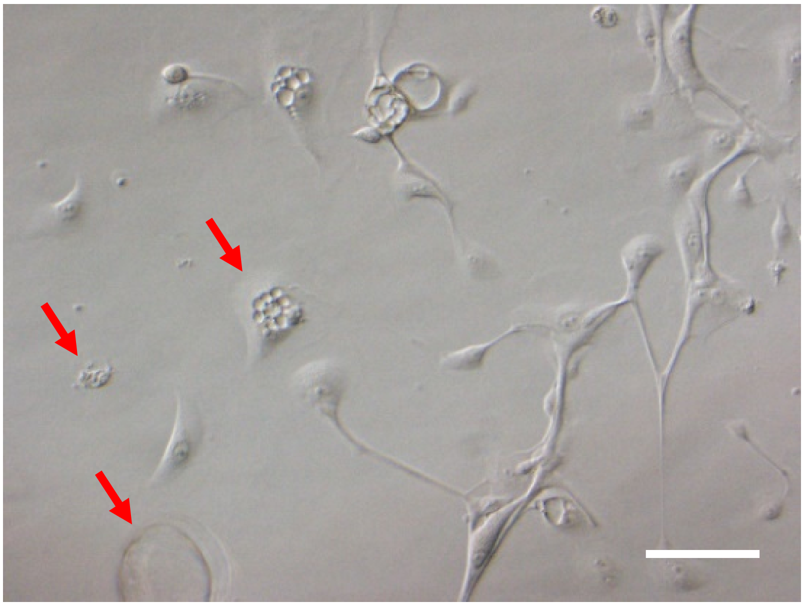

A. KRT5 (green, basal maker)

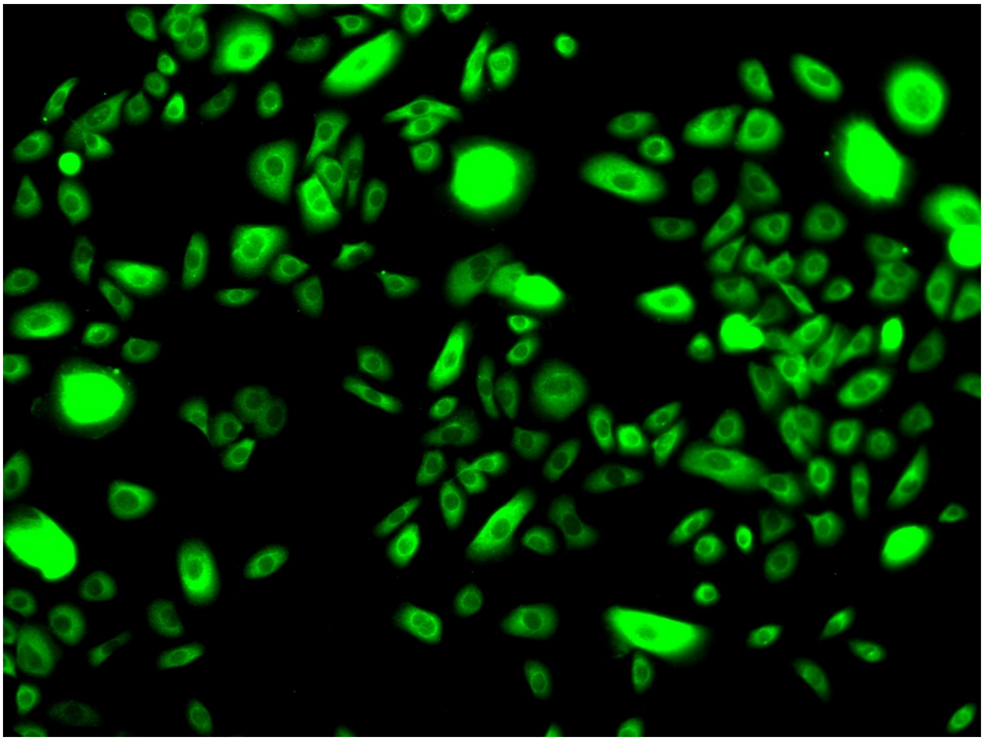

B. KRT5 (green) + Nucleus (blue)

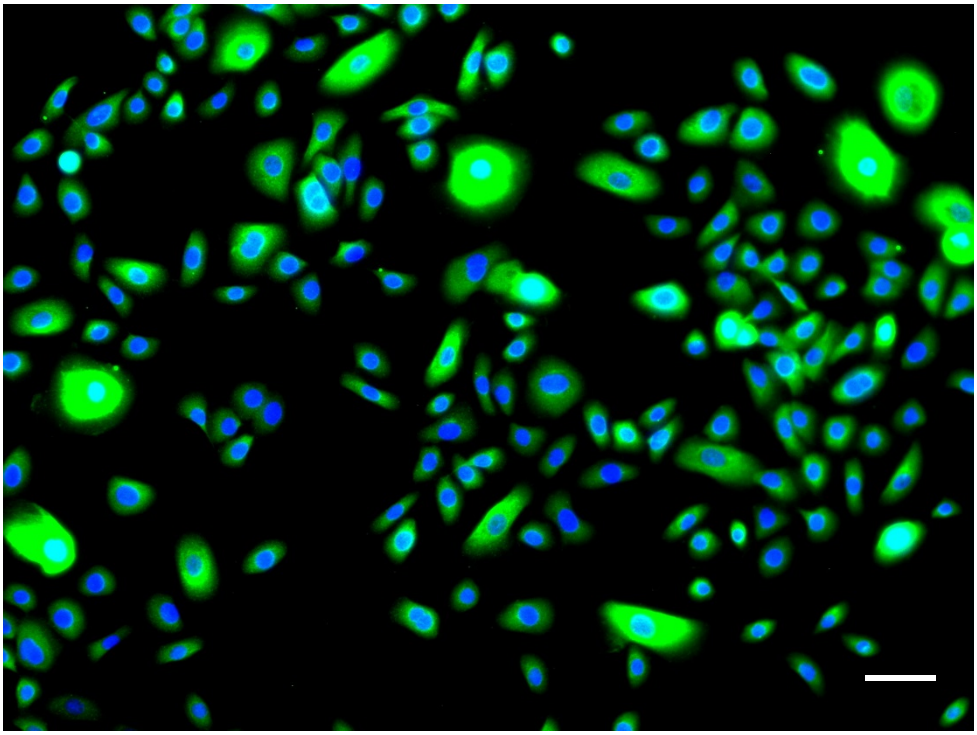

Supplement: Supplementary file 1 — Table S1. List of primers for TaqMan assays. Table S2. List of immunity-related genes among the top 1500 up-regulated genes expressed by hSABCi-NS1.1 on air-liquid interface. Figure S1. Morphology of hSABCi-NS1.1 at late passage. Figure S2. Basal cell marker KRT5 staining in the parental cell prior to immortalization. (PDF 641 kb) [file 12931_2019_1140_MOESM1_ESM.pdf]
